# Vaccination of calves with *Mycobacterium bovis* Bacillus Calmette–Guerin reduces the frequency and severity of lesions of bovine tuberculosis under a natural transmission setting in Ethiopia

**DOI:** 10.1111/tbed.12618

**Published:** 2017-02-06

**Authors:** G. Ameni, K. Tafess, A. Zewde, T. Eguale, M. Tilahun, T. Hailu, A. Sirak, F. J. Salguero, S. Berg, A. Aseffa, R. G. Hewinson, H. M. Vordermeier

**Affiliations:** ^1^ Aklilu Lemma Institute of Pathobiology Addis Ababa University Addis Ababa Ethiopia; ^2^ Armauer Hansen Research Institute Addis Ababa Ethiopia; ^3^ National Animal Health Diagnostic and Investigation Centre Sebeta Ethiopia; ^4^ School of Veterinary Medicine University of Surrey Surrey UK; ^5^ Animal and Plant Health Agency New Haw Surrey UK

**Keywords:** BCG vaccination, bovine tuberculosis, natural transmission

## Abstract

Bovine tuberculosis (bTB) is highly prevalent in intensive dairy farms of the urban “milk‐sheds” in Ethiopia, and vaccination could be a cost‐effective disease control strategy. In the present study, the efficacy of Bacillus Calmette–Guerin (BCG) to protect against bTB was assessed in Holstein–Friesian calves in a natural transmission setting. Twenty‐three 2‐week‐old calves were subcutaneously vaccinated with BCG Danish SSI strain 1331, and matched 26 calves were injected with placebo. Six weeks later, calves were introduced into a herd of *M. bovis*‐infected animals (reactors) and kept in contact with them for 1 year. In vitro and in vivo immunological tests were performed to assess immune responses post‐vaccination and during exposure. Successful vaccine uptake was confirmed by tuberculin skin test and IFN‐γ responses in vaccinated calves. The kinetics of IFN‐γ responses to early secretory antigen target 6 and culture filtrate protein 10 (ESAT6 and CFP10, respectively) and tuberculin skin test responses post‐exposure suggested that the animals were infected early after being placed in contact with the infected herd as immunological signs of infection were measurable between 2 and 4 months post‐initial exposure. Protection was determined by comparing gross and microscopic pathology and bacteriological burden between vaccinated and control calves. BCG vaccination reduced the proportions of tissues with visible pathology in vaccinates compared to control calves by 49% (*p* < .001) with 56%, 43%, 72%, and 38% reductions in the proportion of lesioned tisues in head, thoracic, abdominal lymph nodes, and lungs, respectively (*p*‐values .029–.0001). In addition, the lesions were less severe grossly and microscopically in vaccinated calves than in non‐vaccinated calves (*p* < .05). The reduction in the overall incidence rates of bTB was 23%, 28%, and 33% on the basis of the absence of gross pathology, *M. bovis* culture positivity, and histopathology, respectively, in vaccinated animals. In conclusion, BCG vaccination reduced the frequency and severity of the pathology of bTB significantly, which is likely to reduce onwards transmission of the disease.

## 1 Introduction

Ethiopia, with over 90 million people, is a typical example of the demographics in most developing countries in Africa and Asia with the human population increasing by 3.2% per year (CSA, 2011), leading to increased demand for food production. Hence, cereal crop production has been prioritized at the cost of grazing land for livestock (Tschopp et al., 2010), leading to overstocking and overgrazing of that land, thereby compromising the development in the Ethiopian livestock sub‐sector. For this reason, intensification of livestock production is considered to be the best option. Thus, the number of intensive dairy farms in and around urban centres is increasing. These emerging dairy farms hold cattle breeds optimized for increased milk production, as the milk production potential of indigenous zebu breeds is by far lower than that of either Holstein–Friesian or crosses between these exotic breeds and indigenous zebu breeds. Moreover, as the extensive cattle husbandry management of the Ethiopian farmers cannot satisfy the milk demand of the growing population, the government is encouraging the establishment of intensive dairy farms by the private sectors. However, both the increase in the number of exotic breeds and the intensification of dairy farming are associated with increased prevalence of cattle diseases such as bovine tuberculosis (bTB) (Ameni et al., 2007; Cosivi et al., 1998; Firdessa et al., 2012).

BTB is predominantly caused by *M. bovis* and is characterized by the development of granulomatous lesions in the respiratory tract and also in other tissues of the animal. Globally, this disease impacts in three major ways, namely as zoonotic TB in humans; direct economic losses due to reduced livestock productivity; and indirect economic losses due to livestock trade restrictions.

The increased number of more susceptible exotic breeds together with the increased intensification of production demands prioritization of improved bTB control strategies focusing primarily on intensive dairy farms. In developed countries, the control of bTB is based on a test‐and‐slaughter strategy, which would be too costly to be applied on a national level in Ethiopia or in any other developing country. Hence, there is a need for exploring alternative control strategies such as routine testing and surveillance, pre‐movement testing, movement restriction of infected herds, and vaccination, all of which could be combined with better bio‐security and farm hygiene. In the present study, we have evaluated the performance of BCG in protecting cattle against bTB in a natural transmission setting by exposing vaccinated and non‐vaccinated calves to a cattle herd known to be bTB infected, which complements our previous study (Ameni, Vordermeier, Aseffa, Young, & Hewinson, 2010).

## 2 Materials and Methods

### 2.1 Study setting and sources of experimental calves

The experiment was conducted at Sebeta Agro Industry PLC, a private farm located in Sebeta about 20 km south‐west of Addis Ababa, Ethiopia. At the start of this challenge experiment, its dairy herd consisted of 72 Holstein–Friesian cattle or crosses; thereof, with the zebu breed, all animals were positive in the single intradermal comparative tuberculin test (SICTT), as described further below. However, during the course of experiment the number of reactor animals decreased gradually, and to maintain a reactor to sentinel ratio > 1, 15 skin test reactor animals from Holeta Agricultural Research Centre were introduced ~6 months into the challenge experiment.

Dairy farms located around Addis Ababa were randomly selected and tested for bTB using the SICTT. Thereafter, the experimental calves were recruited from seven bTB negative farms located around Addis Ababa. A total of 49 Holstein–Friesian calves were recruited for the experiment. The calves were allocated to experimental and control groups randomly using a lottery system. The allocation was carried out in batches of 10 calves, as all calves could not be recruited at once. Twenty‐three calves were allocated into a vaccinated group while the remaining 26 calves were used as controls. All the calves were tested negative for bTB when tested with the Bovigam IFN‐γ test prior to their recruitment into the experiment. The experiment was approved both by Ethics Committee of the Armauer Hansen Research Institute and by the Ethical Review Board at the Animal and Plant Health Agency.

### 2.2 Vaccination schedule of the neonates

All calves of the vaccinated group were vaccinated within 2 weeks of birth by subcutaneous injection with 1–4 × 10^6^ CFU BCG Danish SSI 1331(Staten's Serum Institute [SSI], Copenhagen, Denmark), which was supplied as freeze‐dried preparation and reconstituted in Sauton's medium as per the manufacturer's instruction. Until calves were introduced into the reactor herd, at 2 months of age, they were kept isolated in a communal calf pen. During this time, the calves were fed with milk, from PPD‐negative cows, concentrate, and grass on the basis of their age. At around 6 weeks post‐vaccination (when they were about 2 months old), the calves were moved to the bTB‐positive herd and kept in contact with reactor animals for about 1 year.

### 2.3 SICTT in calves

Experienced veterinarian who was blinded with regard to the vaccination status of each calf performed the SICTT. The test was performed four times: at sixth week post‐vaccination and prior to exposure to infected cows, and after 4, 8, and 12 months of exposure to reactors. On each occasion, 0.1 ml avian purified protein derivative (APPD, 25,000 IU/ml, Thermo Fisher, Lelystad, The Netherlands) was injected into the skin intradermally in the cranial part of the neck while 0.1 ml bovine purified protein derivative (BPPD, 20,000 IU/ml, Thermo Fisher) was injected intradermally in the caudal part of the neck, and the increases in skin thickness were measured 72 hr post‐PPD administrations. Results were interpreted according to the recommendations of the World Organization for Animal Health (OIE, 2009).

### 2.4 Whole‐blood culture and IFN‐γ test

IFN‐γ test was performed on animals on a regular basis to monitor the outcome of vaccination as well as the outcome of the exposure to the reactors. Blood samples were collected from the jugular vein into heparinized vacutainers and then transported to the laboratory for stimulation within 8 hr of collection. For stimulation, 250μl of whole blood was dispensed in duplicate into 96‐well flat‐bottom culture plates. Then, 25‐μl aliquots of mycobacterial antigens were added into each well to give the final assay concentrations of 10μg/ml of APPD, 10μg/ml of BPPD, and 10μg/ml of ESAT6/CFP10 peptide cocktail (5μg each peptide/ml). Lectin from *Phytolacca americana* (pokeweed; Sigma) at 5μg/ml and saline (both 25μl) were used as positive and negative controls, respectively. Cultures were incubated at 37°C in a humid 5% CO_2_ atmosphere for 48 hr, and supernatants were harvested and frozen. IFN‐γ in the supernatants were measured by an enzyme‐linked immunosorbent assay using Bovigam test kit (Prionics, Schlieren, Switzerland) in accordance with the manufacturer's instructions.

### 2.5 Post‐mortem examination and pathology scoring

At the end of one‐year exposure period, the calves were transported to the post‐mortem room at the National Animal Health Diagnostic and Investigation Centre (NAHDIC), which is located close to the experiment site. The calves were euthanized humanely and examined for gross lesion of bTB by a veterinary pathologist and two additional veterinarians with expertise on bTB. All three experts were blinded to the vaccination status of the calves. Up to four calves were slaughtered per day and subjected to detailed post‐mortem examination. The lungs and lymph nodes (LNs) were removed for investigation of TB lesions. All seven lobes of the lungs were inspected at the surface and palpated for the presence of TB lesions within the internal parenchyma. Each lobe was then sectioned into 1–2‐cm thick slices to facilitate the detection of TB visible lesions (VL). Similarly, LNs, including the left and right parotid, left and right mandibular, left and right lateral retropharyngeal, left and right medial retropharyngeal, cranial and caudal mediastinal, left and right bronchial, hepatic, and mesenteric LNs, as well as left and right tonsils, were sliced into thin sections and inspected for the presence of VLs. When gross lesions suggestive of bTB were found in any of the tissues, the animal was classified as “VL.” Any animal in which TB‐like lesion(s) were not found was classified as “NVL” (none visible lesions). The severity of gross lesions was scored by a semi‐quantitative scoring procedure as previously described by Vordermeier et al. (2002). Briefly, lesions in the lobes of the lungs were scored separately as follows: 0, no visible lesions; 1, no gross lesions but lesions apparent on slicing of the lobe; 2, fewer than five gross lesions; 3, more than five gross lesions; and 4, gross coalescing lesions. The scores of the individual lobes were added up to calculate the lung score. Similarly, the severity of gross lesions in individual LN was scored as follows: 0, no gross lesion; 1, a small lesion at one focus (just starting); 2, small lesions at more than one focus; and 3, extensive necrosis. Individual LN scores were added up to calculate the total LN score for each LN/tissue category. Finally, LN and lung pathology scores were added together to give the total pathology score per animal.

### 2.6 Histopathological examination and grading of granuloma

For histopathology examination, tissue samples from organs displaying TB‐like gross lesions were collected and immersed in fixative (10% neutral buffered formalin) for 7 days before being processed and embedded in paraffin wax. Four‐micron sections were cut and routinely stained with haematoxylin and eosin (H&E) and Ziehl–Neelsen (ZN) for the detection of TB granuloma and acid‐fast bacilli (AFB), respectively. Slides were examined by light microscopy to determine the distribution of granuloma development stages as defined by Wangoo et al. (2005). Thus, these four stages of granulomas were quantified and analysed as previously described (Aranday‐Cortes et al., 2013). Briefly, Stage I (initial) granulomas comprised clusters of epithelioid macrophages (MΦs), few neutrophils, and occasional Langhans' multinucleated giant cells (MNGCs). Stage II (solid) granulomas were more regular in shape and surrounded by a thin and incomplete capsule. The cellular composition was primarily epithelioid MΦs, Langhans' MNGCs giant cells present, and some infiltration of lymphocytes and neutrophils. Necrosis was minimal or not present. Stage III (necrotic) granulomas were all fully encapsulated with central areas of necrosis. The necrotic centres were surrounded by epithelioid MΦs and Langhans' MNGCs, and a peripheral zone of MΦs, clustered lymphocytes, and isolated neutrophils extended to the fibrotic capsule. Stage IV (mineralized) granulomas were completely surrounded by a thick fibrous capsule and displayed central areas of caseous necrosis with extensive mineralization. The central necrosis was surrounded by epithelioid MΦs and Langhans' MNGCs cells with a peripheral zone of MΦs and dense clusters of lymphocytes just inside the fibrous capsule. They were frequently multicentric, with several granulomas coalescing. The total number and developmental stage of granulomata within each slide were counted.

### 2.7 Isolation of mycobacteria

Isolation of mycobacteria from the LNs and lung tissues was performed in accordance with OIE protocols (2009). Briefly, tissue specimens were collected into sterile universal bottles in 5 ml of a 0.9% saline solution and then transported to the laboratory for bacterial isolation. Individual LN and lung samples were cultured. In VL cases, two or three tissues with lesion were processed for culturing per individual calf. In NVL cases, sections of eight LNs (left and right lateral retropharyngeal, left and right medial retropharyngeal, cranial and caudal mediastinal, and left and right bronchial) were cultured per individual calf. The LNs were sectioned into pieces with sterile blades and then homogenized with a pestle and a mortar. The homogenate was decontaminated by adding an equal volume of 4% NaOH and centrifuged at 1,106 *g* for 15 min. The supernatant was discarded, while the sediment was neutralized with 1% (0.1 N) HCl with phenol red as an indicator. Neutralization was achieved when the colour of the solution changed from purple to yellow. Thereafter, 0.1 ml of suspension from each sample was spread onto duplicate slants of Lowenstein–Jensen medium (OIE, 2009); one was enriched with sodium pyruvate, while the other was enriched with glycerol. Cultures were incubated aerobically at 37°C for up to 8 weeks with weekly observation for growth of mycobacterial colonies. Any culture positive sample was stained by ZN staining, and the presence of isolates was confirmed by the demonstration of acid‐fast bacilli (AFB) in the smears of colonies. Heat‐killed AFB positive samples were investigated by multiplex PCR for the presence or absence of RD4 (Firdessa et al., 2012), a chromosomal deletion that defines *M. bovis*.

### 2.8 Statistical analysis

To estimate the incidence of bTB per group, the number of calves that developed TB was divided by the total number of calves in the group. The efficacy of the vaccine was estimated using the formula described by Orenstein et al. (1985), which considers the incidence rates of the disease in question in the vaccinated and in unvaccinated calves; i.e., the efficacy of a vaccine is the percentage reduction in the incidence rate of a disease among vaccinated calves as compared to the incidence rate in unvaccinated calves. The formula used for calculating vaccine efficacy (VE) in this study was VE = (ARU‐ARV/ARU) ×100%, where ARU is attack (incidence) rate in the unvaccinated group and ARV is the attack (incidence) rate in the vaccinated group. Chi‐squared (χ^2^) test was used to compare the percentages of gross pathology in different tissues of vaccinated and control calves. In addition, the incidence rates of bTB and efficacies of BCG in vaccinated and control calves were compared using χ^2^ test. Comparisons of severity of gross lesion between tissues of vaccinated and control calves were made using non‐parametric *t* test with Mann–Whitney comparison post‐test. Means of optical density at 450 nm (OD450) values for the IFN‐γresponses in the vaccinated and control groups were compared using unpaired *t* test with Welch's correction. In addition, the means of change in skin thickness following skin test in vaccinated and control calves were compared using unpaired *t* test with Welch's correction. A χ^2^ test for trend statistical analysis was performed to compare the distribution of Stage I–IV lesions in vaccinated and control groups, using Prism 6.0 (GraphPad, San Diego, CA, USA). Statistical significance was fixed at *p* < .05.

## 3 Results

### 3.1 Immune responses post‐vaccination

**Figure 1:**
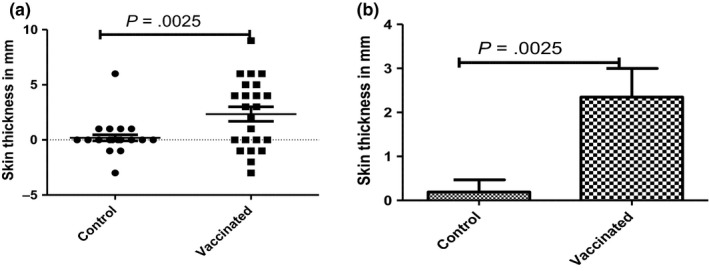
Means of change in skin thickness (mm) following the single intradermal comparative tuberculin test (SICTT) in vaccinated and control calves at sixth week post‐vaccination with BCG. Skin thickness as shown individually (a) and in group (b) was significantly higher in vaccinated calves than in control calves (*p* = .0025)

The SICTT and IFN‐γ responses to APPD and BPPD were investigated in BCG‐vaccinated and control calves following vaccination. In the vaccinated group, IFN‐γ responses to BPPD started to rise 1 week after BCG vaccination, peaked at the third and fourth week post‐vaccination (data not presented). In addition, all calves were SICTT tested 6 weeks post‐BCG vaccination. As expected, the SICTT responses in the vaccinated calves were statistically significantly increased compared to the control animals (Figure 1a/b, *p* = 0.003; unpaired *t* test) that all tested SICTT negative. Taken together, the IFN‐γ release assay and SICTT result post‐vaccination demonstrated that the BCG vaccination induced a cellular immune responses confirming vaccine uptake.

### 3.2 Immune responses after vaccinated and control calves were exposed to infected herd

**Figure 2:**
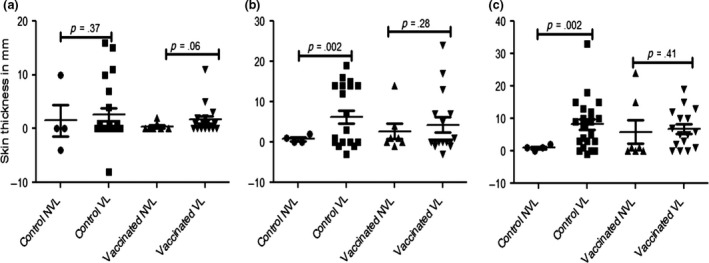
Single intradermal comparative tuberculin testing (SICTT) responses in vaccinated and control calves with visible (VL) and non‐visible lesions (NVL) The calves were tested with SICTT at the fourth (a), eighth (b) and 12th (c) month post‐exposure to infected herd. The means of change in skin thickness following SICTT were significantly greater (unpaired *t* test with Welch's correction) in control calves with VL than in control calves with NVL at eighth (*p *= .002) and 12th (*p* = .002) month post‐exposure to infected herd. However, although the mean of change in skin thickness after SICTT was greater in vaccinated calves with VL than in vaccinated calves with NVL at all the months tested, the differences were not significant at any of the months

The kinetics of skin test (SICTT) and IFN‐γ responses were determined during the 12 months in‐contact period of BCG‐vaccinated and control calves with the *M. bovis*‐infected herd. The results were stratified according to the presentation of visible gross pathology at the slaughter of the calves, i.e., the presence or absence of visible lesions (VL or NVL, respectively). SICTT responses were determined at month 4, 8, and 12 of the in‐contact period. The results are shown in Figure 2. SICTT responses increased in VL control animals after 4 months exposure compared to NVL animals, although this difference was not yet statistically significant (Figure 2a). However, these responses were significantly higher at months 8 and 12 in VL controls compared to their NVL counterparts (Figure 2b/c, *p* = .002 and .002, respectively). Indeed, no SICTT responses were induced in the four NVL animals at months 8 and 12 (Figure 2b/c). SICTT responses in VL BCG‐vaccinated calves were elevated at months 4, 8, and 12 of the exposure period compared to their NVL group mates. Differences between VL BCG and VL control animals were not statistically significant.

**Figure 3:**
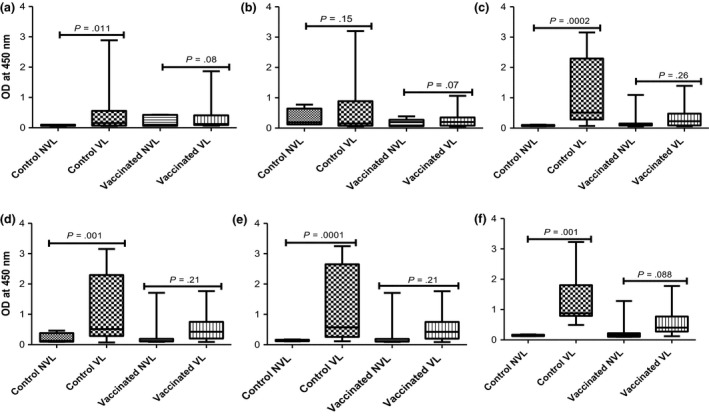
Kinetics of IFN‐γ response to ESAT6/CFP10 peptide cocktail in vaccinated and control calves with visible lesions (VL) and non‐visible lesions (NVL) groups at various time points post‐exposure to infected herds for one year. The means of IFN‐γ response estimated by optical density (OD) measured at 450 nm were monitored for 12 months after the calves were exposed to the infected herd. The means of the IFN‐γ responses are compared (unpaired *t* test with Welch's correction) in control calves with VL and in control calves with NVL at the second (a; *p* = .011), fourth (b; *p* = .15), sixth (c; *p *= .0002), eighth (d; *p *= .001), 10^th^ (e; *p *= .0001) and 12^th^ (f; *p* = .001) month post‐exposure to the infected herd. However, although vaccinated calves with VL demonstrated relatively stronger IFN‐γ responses than vaccinated calves with NVL at the different months of exposure, the difference between these two groups was not significant at any of the months

The IFN‐γ responses towards stimulation with the RD1 antigens ESAT‐6 and CFP‐10 were determined in vaccinated and control calves 2, 4, 6, 8, 10, and 12 months after they were placed in contact with the infected herd (Figure 3). These antigens are not recognized after BCG vaccination, and their responses can be used to monitor *M. bovis* infection. IFN‐γ responses in VL control calves were signficantly elevated compared to NVL controls at all time points (Figure 3a, c–f) except when measured 4 months after exposure (Figure 3b). Similarly, increased IFN‐γ responses in VL compared to NVL animals were observed in the BCG‐vaccinated animals when this parameter was determined at 4 to 12 months of the exposure period (Figure 3b–f), although due to one outlying response, these differences were not or not quite statistically significant (*p* values of 0.07 to 0.26, Figure 3). The data also suggested that IFN‐γ responses of VL BCG animals were lower at all time points tested compared to VL control animals (Figure 3b–f) and developed later than in unvaccinated calves (Figure 3a), although these differences were not statistically signficant.

Taken together with the corresponding SICTT data, the IFN‐γ responses displayed by VL control animals were suggestive that immune responses demonstrating *M. bovis* infection were detectable from 2 (IFN‐γresponses) to 4 months (SICTT) of exposure to the infected herds.

### 3.3 Efficacy of BCG in protecting against bovine tuberculosis: full protection

To determine whether BCG vaccination imparted full protection against bTB disease, we first determined the presence or absence of visible, grosspathology (VL or NVL, respectively), microscopic pathology, or the presence of *M. bovis* in tissues (Table 1). In the unvaccinated group, 85% (22/26) of calves presented as VL had microscopic lesions typical of bTB and contained *M. bovis* in post‐mortem tissues (Table 1). By contrast, 65% (15/23), 57% (13/23), and 61% (14/23) of the vaccinated calves presented gross pathology, microscopic pathogy, or were *M. bovis* culture positive, respectively (Table 1). These figures equate to 23%, 33%, or 28% protection, respectively, when these three parameters are taken into account. While only the difference in the frequency of microscopically lesioned calves is statistically signficant (*p* = 0.031), the data demonstrated a consistent degree of protection against bTB disease following BCG vaccination.

**Table 1:** Efficacy of BCG in protecting against bTB

	Vaccinated group (23)		Control group (26)		Attack rate in vaccinated calves	Attack rate in control in calves	Efficacy
	Positive	Negative	Positive	Negative			
Gross pathology	15	8	22	4	65%	85%	23%
Culture	14	9	22	4	61%	85%	28%
Histopathology	13	10	22	4	57%	85%	33%

### 3.4 Distribution, frequency, and severity of typical bTB lesions in vaccinated and control calves

**Figure 4:**
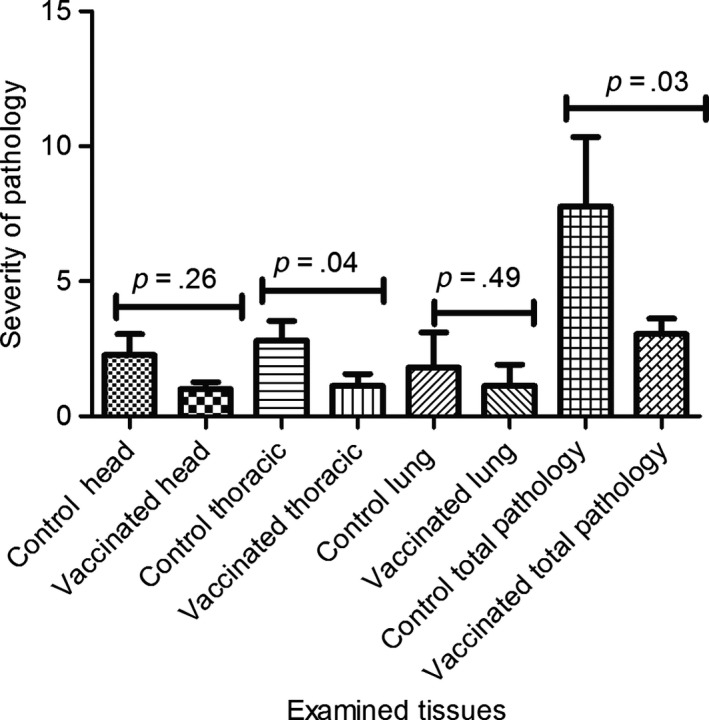
Severity of gross pathology of bTB in different tissues of vaccinated and control calves. Mann–Whitney test was used to compare the severity of pathology between vaccinated and control calves in different tissues. The pathology was more severe (*p* = .04); in the thoracic lymh nodes of control calves than in the thoracic lymph nodes of vaccinated calves; total pathology was more severe (*p *= .03) in tissues of control calves than in tissues of vaccinated calves

Further assessment of BCG protection against bTB was carried out by determining the reduction in the severity and within animal dissemination of disease. The severity of pathology was scored according to Vordermeier et al. (2002) in post‐mortem tissues of BCG‐vaccinated and non‐vaccinated calves and was compared as presented in Figure 4. The pathology was more severe (*p* = 0.04; Mann–Whitney test) in the thoracic lymph nodes of control calves (median ± *SD*, 1.5 ± 3.72) than in the thoracic lymph nodes of vaccinated calves (0.0 ± 1.13). While we observed similar reductions in the severity of gross pathology in vaccinated calves also in head lymph nodes and the lungs compared to unvaccinated controls, these reductions did not reach statistical significance (Figure 4). However, the total pathology scores that integrate the pathology scores of the different tisses were significantly lower in vaccinated as compared to control calves (Figure 4, *p* = 0.03; control calves scores = 5.0 ± 13.12; vaccinated calves = 3.0 ± 2.15).

The protective effect of BCG vaccination on reducing gross pathology was also confirmed when the frequencies of visible lesions found in the examined tissues were compared between control and BCG‐vaccinated calves (Table 2). The percentages of lesioned tissues found in the head, thoracic, and abdominal LNs, as well as in the lungs, were signficantly reduced by 56%, 43%, 72%, and 38%, respectively, when vaccinated calves were compared with the control calves (Table 2; *p* < .03 to <.0001). Overall, this represents a reduction in lesioned tissues by 49% (Table 2, *p* < .001). The data presented in this section, therefore, confirm a clear and strong protective effect of BCG on reducing the severity of the consequences of *M. bovis* infection.

**Table 2:** The percentageof TB macroscopic lesions in BCG vaccinated and control calves after exposure to an infected herd in a natural transmission setting

Tissue	BCG vaccinates Proportion of lesioned tissues	Controls: Proportion of lesioned tissues	Reduction in frequency of pathology	Chi‐square test	*p*‐value
Head lymph nodes (LN)	12.5% (23/184)	28% (59/208)	56%	14.86	.0001
Thoracic LNs	32% (37/115)	56% (73/130)	43%	14.18	.0002
Abdominal LNs	11% (5/46)	38% (20/52)	72%	9.78	.0017
Lung lobes	16% (26/161)	26% (47/182)	38%	4.77	.029
Total Pathology	18% (91/506)	35% (202/572)	49%	40.74	.0001

### 3.5 Assessment of microscopic bTB lesions in vaccinated and control calves

**Figure 5:**
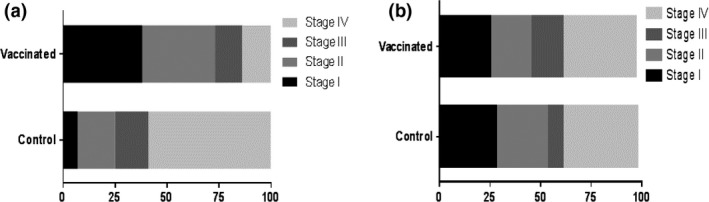
Frequency of occurrence of the different stages (I–IV) granulomas within the head, neck (a), and thoracic lymph nodes (b) of vaccinated and control calves. The bars represent the percentage of granulomas within each developmental stage. A significant difference was observed in the frequencies of lesions in the head and neck lymph nodes of the vaccinated and control groups (panel a, *p* < 0.05), but not in the frequencies of the thoracic lymph nodes (b) between the vaccinated and control groups

Tissues presenting with TB‐like lesion upon gross pathological examination were further subjected to microscopic histopathological analysis. The distribution, frequency, and severity of microscopic lesions were assessed, and the results are depicted in Figure 5. Microscopic lesions were scored from Stage I to Stage IV according to the classification system by Wangoo et al. (2005).

More than 500 individual granulomata were scored. The majority of granulomata were observed in the thoracic LNs (cranial mediastinal, caudal mediastinal, cranial tracheobronchial, and left and right bronchial) and the “head and neck” LNs (left and right parotid, and mandibular and medial and lateral retropharyngeal). As only a very low number of granulomata were observed in other tissues such as the lung, liver, hepatic or mesenteric LNs, statistical analyses were only performed using the data generated by scoring granulomata in the head and neck LNs (Figure 5a) and thoracic LN (Figure 5b). When the head and neck LNs were assessed, the distribution of granulomata of different development stages was statistically significantly different (*p* < .05) between vaccinated and control calves, with a distinct enrichment of early stage (I and II) granulomata in the vaccinated animals compared to controls in whom granulomata of the most developed granuloma stage (IV) dominated (Figure 5a). By contrast, no statistically significant difference between vaccinates and controls was observed in the distribution of granuloma developmental stages in the thoracic LNs (Figure 5b).

## 4 Discussion

In the present study, the efficacy of the BCG vaccine was evaluated in Holstein–Friesian calves in a natural transmission setting. When cellular immune responses were determined in vaccinated calves before they were introduced into the infected herds, neither IFN‐γ nor tuberculin skin test responses correlated with protection determined by post‐mortem at the end of the in‐contact period (VL versus NVL calves, data not shown). This therefore confirms that these two parameters are poor predictors of protection.

Based on the development of ESAT‐6‐/CFP‐10‐specific IFN‐γ responses following the exposure of calves to the infected herd, it can be hypothesized that the infection events took place during the first 2–4 months of calves being in contact with infected animals. Interestingly, ESAT‐6‐/CFP‐10‐induced IFN‐γ responses developed at a slower pace in vaccinated VL animals compared to VL control calves. This could be because BCG vaccination reduced the extent of pathology or led to a slower disease progression compared to naïve calves. Either hypothesis is supported by earlier data, demonstrating that the extent of in vitro IFN‐γ production after stimulation with ESAT‐6 directly correlated with the extent of pathology (Vordermeier et al., 2002).

The efficacy of BCG in protecting against *M. bovis* infection under a natural transmission setting was estimated using either gross pathology, microscopic lesion of bTB, or isolation of *M. bovis* as interpretation criteria. In the present study, the efficacy of BCG for protection against disease was low, whereas its effect on reducing the extent of pathology was significant. The average efficacy of BCG to protect fully against bTB recorded in this study was around 30%. This rate is considerably lower than the efficacy reported earlier by similar studies conducted in Ethiopia (Ameni et al., 2010) and in Mexico (Lopez‐Valencia et al., 2010). This difference in full protection between the present and the previous Ethiopian natural transmission study (Ameni et al., 2010) could be attributed to differences in the severity of bTB in the infected reactor herds that served as sources of infection: Although similar ratios of calves to reactor cows were maintained in both experiments, the severity of disease was heavier in the herd used for the present study compared to the herd used for the previous study. For example, overt clinical signs of bTB were more prevalent in the infected herd used in the present study. Nevertheless, both the present and the earlier studies (Ameni et al., 2010) results are within the efficacy range of BCG (0% and 75%) reported from experimental studies and trials in cattle conducted by different researchers in different countries between 1959 and 2002 (reviewed by Hewinson, Vordermeier, & Buddle, 2003). Thus, the result of the present study may be reflective also of the inherent variability of BCG to impart protection at population and individual animal levels.

Nevertheless, in the present study, a significant level of protection by BCG vaccination could be demonstrated based both on reduction in the number of tissues with visible pathology and on the reduction in granulomata severity. Thus, the significant reduction in pathology following BCG vaccination could lead to a reduced onward transmission rate from vaccinated cattle to other susceptible cattle. This indirect vaccination effect would therefore very likely contribute to a reduction in the prevalence of bTB from vaccinated herds, as had been shown in an earlier trial in the UK (Doyle & Stuart, 1958). For example, BCG vaccination could reduce the incidence of bTB in vaccinated herds to a level where more conventional test‐and‐slaughter approaches could be affordable. The reduction in the development of granulomas further confirms the finding of BCG imparting protection by reducing pathology. From the disease transmission point of view, this has great epidemiological implication, as lesions that are confined with bacilli contained in granulomas at certain anatomical sites may also prevent disease transmission to other animals (Johnson, Spencer, Hewinson, Vordermeier, & Wangoo, 2006).

## 5 Conclusion

In conclusion, although the reduction in the proportion of susceptible animals through BCG vaccination (full protection, direct effect of vaccination) was lower than that recorded in the previous study, the significant reduction in the severity and distribution of visible and microscopical pathology in vaccinated calves is likely to reduce onward transmission to other animals (a so‐called indirect vaccine effect). This would have a beneficial impact on disease control in vaccinated farms.

## Conflict of Interest

Authors declare that there is no conflict of interest.
